# Picture analysis of billboards and infographic graphics advertising COVID-19 on promoting preventive behaviors and taking vaccination against the Coronavirus disease pandemic

**DOI:** 10.1038/s41598-024-56758-z

**Published:** 2024-03-15

**Authors:** Fereshteh Mohamadpour, Ardalan Askarian, Mehrdad Askarian

**Affiliations:** 1 Department of Community Medicine, School of Medicine, Shiraz University of Medical Sciences, Shiraz, Iran; 2 College of Arts and Science, University of Saskatchewan, Saskatoon, Canada

**Keywords:** COVID-19, Billboard, Infographic, Public attention, Prevention, Vaccination, Public health, Disease prevention, Preventive medicine

## Abstract

Today, advertising science is a tool that helps advertisers to design their advertising to meet the needs of the audience. In this regard, knowing and understanding the audience is one of the most important points that advertisers should pay attention to before advertising in order to better attract the audience. This study has been done with the aim of billboards and infographics analysis related to promoting preventive behaviors and vaccination against the Coronavirus disease pandemic and investigating the opinion of the general adult population of Iran. The method used in this research is the qualitative method. In this research, according to the type of data and research goals, Kress and Van Leeuwen’s discourse theory method has been used. The sample size includes 36 advertising billboards and infographics. Data collection has been done through searching the sites and websites of health networks and medical education centers in Iran, taking pictures of infographics and billboards in public places, and also receiving archive files of pictures from the public relations of health networks and medical services. The data was collected from February 19, 2020 to December 30, 2022 (the time frame of the pandemic and public vaccination program in Iran). Then, an online survey about promoting preventive behaviors and taking vaccination against the Coronavirus disease pandemic was designed in SurveyMonkey and its link was provided to the audience through virtual networks and other platforms. The assessment of validity involved experts in infection control and linguistics. The reliability of the measurement, determined through the Cronbach’s alpha internal consistency coefficient, yielded a coefficient of 0.968. In this study, data analysis was conducted using IBM SPSS Statistics software, version 15.0 (IBM Corp., Armonk, NY, USA). Finally, users’ opinions about of billboards and infographics were analyzed using descriptive statistics. The results of component analysis and surveys show that visual components such as «The staring look at the spectator (Demand)», «Head-on Shot (inclusion)», «Down Shot (Creating a sense of participation for the represented person)», «Close-up (intimate/individual relationship)», «Level Shot (equality)» and «High-Angle Shot (Presenting power)» in medical advertising has had a great impact in arousing public opinion to create a positive attitude towards preventive measures and vaccination during the Coronavirus disease epidemic. The results of this research show that in visual communication, visual components play a significant role in creating and maintaining target ideologies. Also, advertising in the field of preventive measures in medical sciences requires certain rules that determine people’s culture and the main foundation of their attitude and thinking. Therefore, it is necessary to know such knowledge and learn it by the medical staff to deal with critical situations.

## Introduction

SARS-CoV-2 is a new epidemic and infectious disease that is mostly transmitted through droplets^1^. The speed of progress of this type of disease around the world in the last few years was such that it severely affected global health^2–5^. Infectious diseases have existed in all historical periods and have intensified over time^6^. SARS-CoV-2 has been one of the main causes of death, social and economic problems, aggravation of mental disorders such as anxiety, stress and depression, as well as the disability of people around the world in the last few years^3–5,7,8^. This disease created very difficult conditions for governments and people^7^. Preventive measures in the health care sector are very important in such situations^9,10^. Organizations in charge of health in different countries have always sought the goal that all people have the possibility of a healthy and quality life and to reduce the gap between the quality of services through health service training and preventive measures^4,5,11^. Empowering people to increase control over their own health and continuously improving the health of the society they live in will promote health^12^. Health promotion includes behaviors and actions through which people will have more control over decisions and actions that affect their health and that of their community^13^. Increasing people’s awareness of the causes and consequences of diseases has a significant contribution in promoting preventive behaviors, which confirms the need to improve knowledge in this field and provide scientific solutions and preventive strategies. Advertising billboards and infographics is one of the most convenient, cost-effective and effective strategies that health centers and medical services around the world use to try to push public opinion to create a positive attitude towards preventive measures and vaccination^14–16^.

So far, researchers have conducted research in various fields regarding the ways to prevent and control the SARS-CoV-2. These researches include topics such as epidemiological research^17,18^, development of candidate therapies^19–21^, characteristics of disease transmission^22,23^, Disease Prevention^24–27^, clinical characteristics of the disease^28,29^, diagnostic methods^30–32^. In recent decades, researchers of various sciences have become increasingly interested in research that investigates the role of language in creating surrounding realities. The set of social and cultural conditions creates the context for the occurrence of text or writing, speech and non-verbal communication in a general proposition^33^. Therefore, language consists of signs and symbols that convey meaning and equip people with concepts that can help them better understand the world around them, think, pay attention to past experiences, and predict future events^34^. Language determines people’s culture and is the main base of attitude and thinking^35^. Few researches have been done in the field of linguistic analysis on the topic of Coronavirus disease epidemic. These researches include topics such as Linguistic and meta-linguistic meanings of words related to the Coronavirus disease^36^, The effects of the Coronavirus disease and the social crisis caused by it, as well as neologisms on language changes^37^, Linguistic Analysis of Fear-Factor Lexemes on Coronavirus disease Pandemic^38^, Reflection on the social and psychological consequences of the Coronavirus Pandemic in the new vocabulary of medical discourse^39^, content analysis of communication strategies and their effects on public engagement on social media^40^, how public confidence on during the COVID-19 pandemic by Chinese media^41^. Also, very few researches have been done on the effect of infographics and posters on persuading people to comply with health principles and protocols and vaccination, as well as increasing public awareness during the Covid-19 epidemic^14–16,42,43^.

In a society where people witnessed the explosion of information on the Internet and other media, the existence of various information about Coronavirus disease (prevention and general vaccination) caused confusion or even created false and incorrect thoughts and backgrounds in the society^44–46^. For this reason, people’s attention was mainly drawn to official government announcements around the world, as well as official opinions of the medical community, so that they could receive reliable information^47^. During the Coronavirus disease epidemic, infographics and billboards served as vital tools to increase public awareness and manage the crisis^14–16^. Despite extensive research on the Coronavirus disease epidemic’s impact, linguistic dimensions and discourse and cognitive structures surrounding the corona crisis have not been thoroughly investigated. Also, humans have special procedures to give meaning to their daily life^48^. In this regard, language is considered as a relationship or mediator of the social world, a very important element^48^. It is necessary and useful to pay scientific and careful attention to the issue of communication with people in the society using different forms of language at micro and macro levels in order to analyze and explain some issues related to medical science. It is very important to conduct research in the field of increasing public attention towards medical advertisements^14^. Since public participation is very necessary to prevent and control the spread of epidemic diseases; Therefore, learning about increasing public attention and knowing such knowledge will help the treatment staff to be more aware and considerate when guiding people to deal with such situations.

Discourse situations form specific discourse processes^33^. The role that linguistic agents and discourses play in these specific situations will influence the possible topics discussed as well as the interpretation provided about people’s social actions^33^. This is also extremely important in medical researches. Providing information in the form of billboards and infographics (multimedia contents) reduces the time it takes for the audience to receive information, and it is much more effective and usable than just written-verbal messages^49^. Considering that the presentation of multimedia contents in informing people is more general, therefore, when producing such contents by medical service centers, it is very important to consider the basic culture of each society. Therefore, the current research aims to investigate the billboards and infographics related to the Coronavirus disease pandemic from a different angle with an interdisciplinary perspective that is a combination of medical science and linguistics.

## Methods

According to the type of data and research objectives, the main method in this research is the qualitative method and type of multimedia discourse analysis and the survey of the audience has been conducted using descriptive statistics method. The method of multimedia discourse analysis investigates other modes of communication such as images, graphs, videos and similar items in the field of social interactions^50^. Multimedia texts are texts that consist of different expression methods such as headlines and titles, photos, videos, and self-written and it creates a semantic context in which the meaning is formed separately in each of these modes of expression and is affected by it^50^. In this direction, specifically, three main categories of “inference about the reference of communication”, “description and inference about the characteristics of communication” and “inference about the effect of communication” are investigated. According to the research goals and more detailed explanation and analysis of billboards and infographics, Kress and Van Leeuwen’s discourse theory^51^ has been used. Kress and Van Leeuwen’s theory is one of the most consistent and codified theories to analyze the meanings represented in the images. Kress and van Leeuwen use the general phrase of participants for the subjects and elements in the picture and for this purpose, they introduce two models of narrative and conceptual representation^51^. The world represented in the image establishes a relationship with the viewer of the image with its specific viewing angle. To study this level of image meaning, Kress and Van Leeuwen suggest three components: contact, distance, and viewing angle^51^. The look at the audience or the type of look represented in the image, and the type of relationship between the look and the audience with the concept of contact, which may contain meanings and states such as mockery, anger, passion, encouragement, etc^51^. By using the distance component, it is also determined how are close or far the depicted people, places and objects^51^. In Kress and Van Leeuwen’s theory, distance has three dimensions, which are: social distance, social relationship and social interaction^51^. The last function, the textual function, deals with how meanings are arranged and integrated within a dynamic text^51^. Visual editing of an image inevitably requires its own special semiotic structure and rhythm^51^. Beginning, middle, end, problem and solution, argument in favor and argument against^51^. For a more detailed analysis of text meta-roles, Kress and Van Leeuwen present three systems of information value, frame and prominence, that their combination and connection with each other will be a precise solution for investigating meta-roles^51^. The information value refers to the location of the elements of an arrangement and according to the left/right, center/margin and top and bottom placement of the elements and in a more general sense, image areas, it gives them specific information^51^. The elements on the left are pre-existing and known and the elements on the right are presented as new elements^51^. Up means being ideal and realistic and down means about providing information with practical and realistic details^51^. The image frame shows that the elements of a composition can have certain distinct identities or be represented interdependently^51^. In other words, the presence or absence of framing connects or separates the elements of an image. This indicates whether they are dependent on each other^51^. Separation is created from methods such as lines and empty spaces between elements or color difference and color contrast^51^. Prominence also includes some elements that can attract attention compared to other elements, and this is also created through foreground or background, relative size, contrast in color strength, brightness, etc^51^. Table 1 shows Kress and Van Leeuwen’s discourse model.

**Table 1:** Cress and Van Leeuwen model^51^.

Elements in the image	Conceptual representation
The staring look at the spectator	Demand
The absence of staring look at the spectator	Presentation
Close-up	Intimate/individual relationship
Medium shot	Creating a social relationship
Long shot	Creating a non-individual relationship
Head-on shot	Inclusion
Dutch angle shot	Separation
High-angle shot	Presenting power to the spectator
Level shot	Equality
Down shot	Creating a sense of participation for the represented person

### Data collection

Data collection has been done through searching the sites and websites of health networks and medical education centers (By entering the link “https://www.larums.ac.ir/university-url” and accessing 64 websites of medical sciences universities in Iran and by searching the keywords “Corona”, “Coronavirus”, “Covid-19” and “SARS-CoV-2” by manual search method; Archives related to preventive measures during the corona epidemic and public vaccination were reviewed, and billboards and infographics that met the conditions for inclusion in the study were selected.), taking pictures of infographics and billboards in public places, and also receiving archive files of pictures from the public relations of health networks and medical services. The data are related to the time frame of the Coronavirus disease pandemic and public vaccination in Iran (from 19 February, 2020 to 30 December, 2022). In this research, 36 advertising billboards and infographics were selected. The samples were selected purposefully. The number of samples has increased to the point where theoretical saturation is done and a new pattern can be extracted from it. In fact, it can be acknowledged that the characteristics of a theoretical class have reached saturation when there is no other data that will cause development and adjustment to the existing theory. In this situation, even if new data is entered into the research, it will not change the classification of the research categories or it will not be a case for creating a new model. In this research, the sampling continued until the exploratory components of the billboards and infographics were not repeated by the researchers and no new theme were raised. The way of selection and criteria for entering and exiting billboards and infographics in the research is as follows: Billboards and infographics according to the written text of advertisements, color, arrangement of elements, two- or three-dimensionality of images, size of writings and shapes, their locations in the city and factors that affect the audience’s attention. Also, the samples were selected by considering the characteristic of having at least one linguistic factor along with advertising and persuasive slogans to analyze. The criterion for choosing the sites from which the billboards and infographics were taken were to be popular and have enough credibility to gain the trust of the audience. For this reason, reliable news sites and public relations sites of scientific centers affiliated to the Ministry of Health, Medicine and Medical Education of Iran were selected as target sites. Since the current research is qualitative, efforts will be made to cover the maximum variety of samples. After data selection and pictures analysis, an online survey form was methodically designed. This survey was crafted utilizing SurveyMonkey, a reputable survey platform. The assessment of validity involved experts in infection control and linguistics. The reliability of the measurement, determined through the Cronbach’s alpha internal consistency coefficient, yielded a coefficient of 0.968. In this study, survey analysis was conducted using IBM SPSS Statistics software, version 15.0 (IBM Corp., Armonk, NY, USA). Subsequently, the survey link was widely disseminated via virtual networks and diverse online platforms to investigate and analyze the opinion of the audience about the pictures. The statistical population includes all strata of society and from the group at risk of SARS-CoV-2 in Iran in the Coronavirus disease and post-Coronavirus disease years. Considering that the number of participants should be representative of the entire community under investigation, therefore, based on Cochran’s formula^52^, the sample size was equal to 350 people. The age group of people was selected from 18 to 70 years. The method of selection and the entry and exit criteria of the participants, according to the type of subject, tried to select them from different strata of the society and from the group at risk of corona virus as far as possible. In order to achieve this goal, the study field included citizens living in all parts of Iran. The criteria for entering people into this research are: Monolingual, the ability to speak Persian, the ability to read and write, not having jobs and disciplines such as literature, writing and teaching, in which case people are considered professional users of speech and language. Exclusion criteria include unwillingness to voluntarily participate in the survey, lack of honest cooperation of participants, bilingualism or multilingualism, professional language user, inability to read and write. Lastly, users’ opinions were analyzed using descriptive statistics.

### Ethical approval and consent to participate

The Ethics Review Board of shiraz university of medical sciences, approved the present study with the following number: IR.SUMS.REC.1402.017. Informed consent was obtained from all subjects.

## Results

After collecting the information, at first, the data analyzed and the understanding of the relationships governing the components and the ideological construction was done based on Cress and Van Leeuwen model^51^. All communication processes are somewhat law-based; Although the nature of these laws may be infinitely diverse. Image components, like words, have wide meanings. The meanings that are conveyed to the audience through images are the meanings that cannot be conveyed through language. Visual communication, like language, plays its role in creating and maintaining the desired ideologies of society, which can serve to create, perpetuate and legitimize some social concepts. Table 2 shows the billboards and infographics of this research.

**Table 2:**
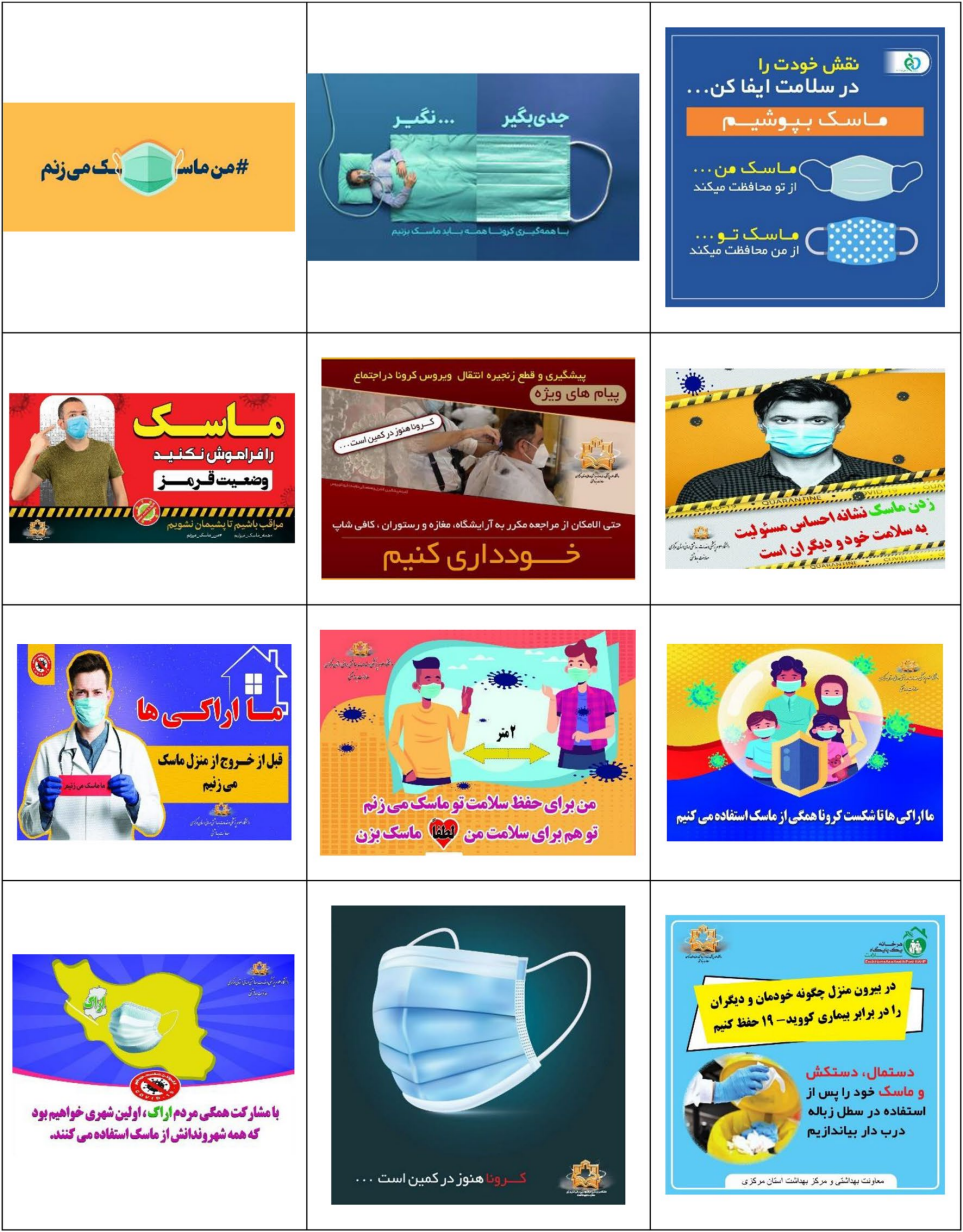

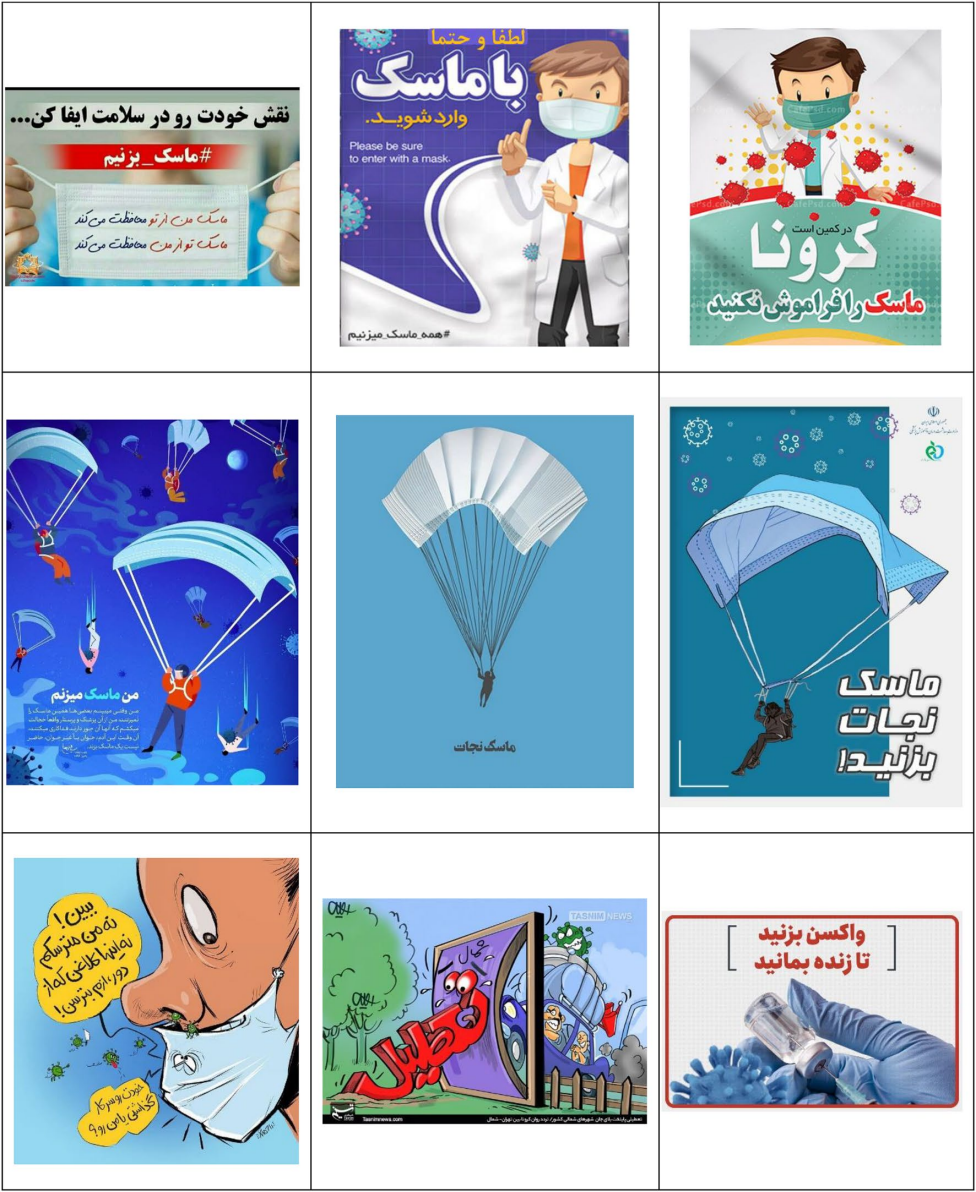

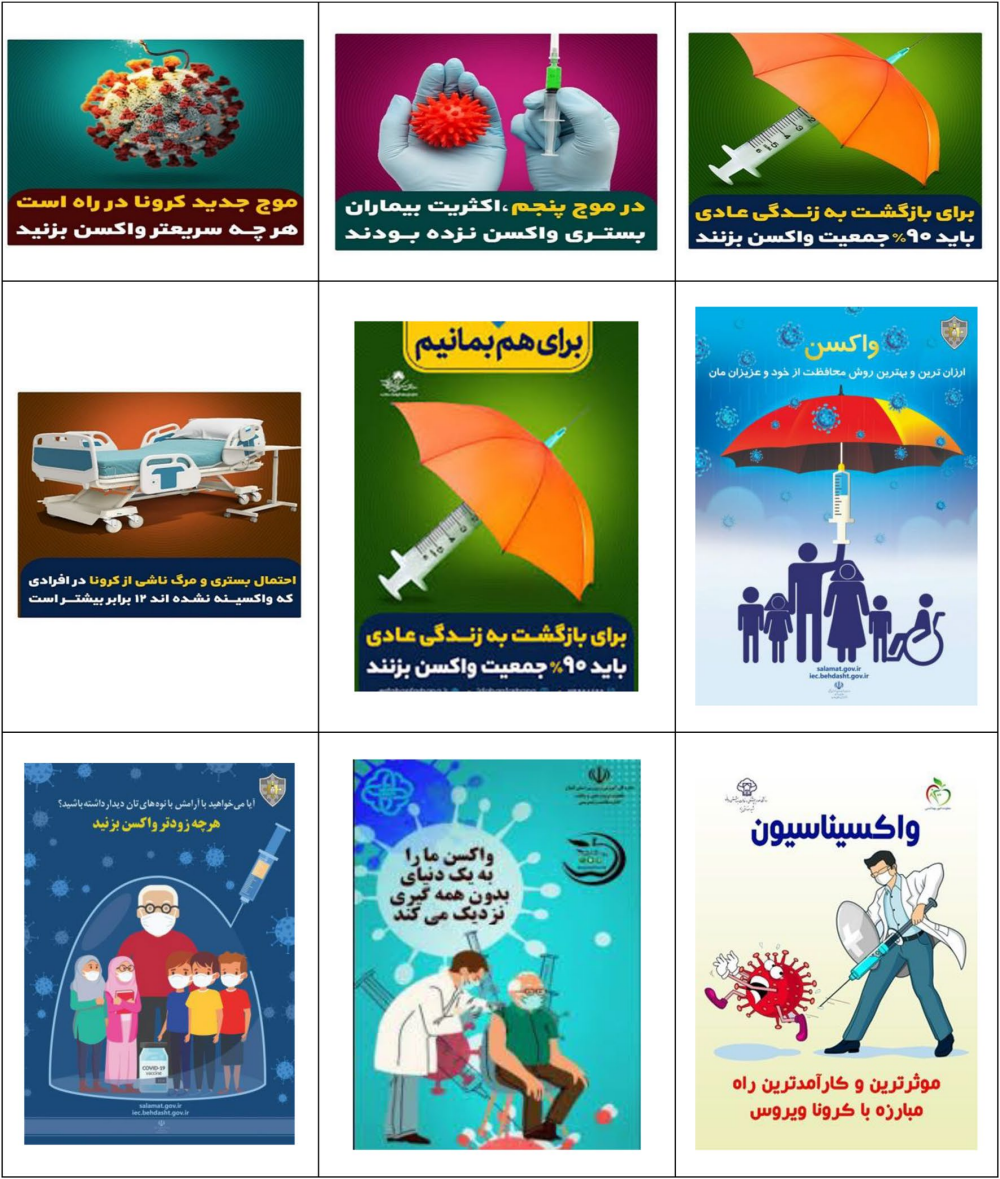

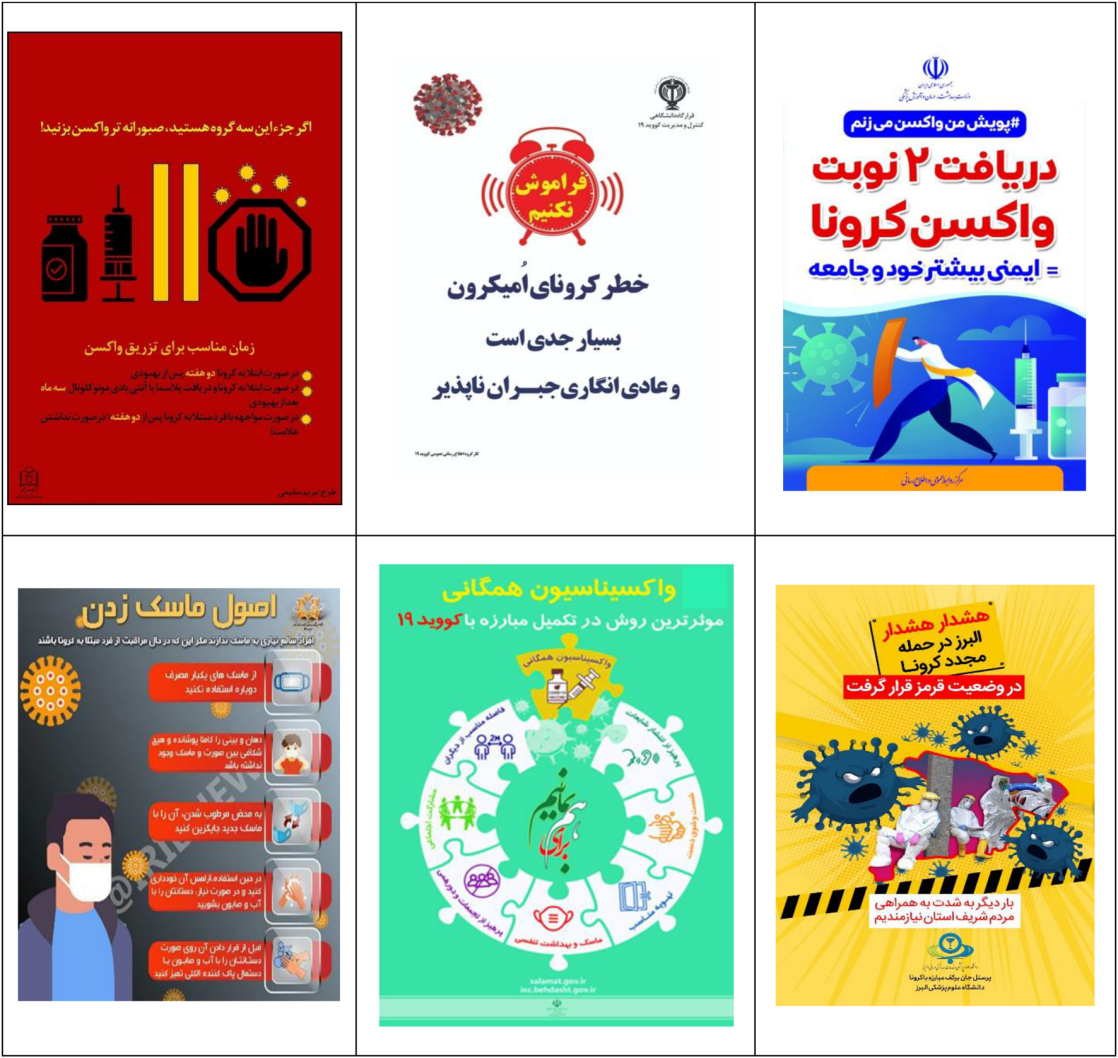
The billboards and infographics.

### Data semiotic analysis based on Kress and Van Leeuwen’s model

#### Types of looks in pictures

The conceptual look is dependent on the object. The field of looking includes the act of seeing, the act of being seen, the act of receiving, and the act of interpretation. In fact, when the audience looks at the object, they are first seen by that object. Therefore, at first, the audience is called by the look of the object and is invited to look with the look of the object^53^. Types of looks from the point of view of Kress and Van Leeuwen, it is: the staring look at the spectator, which indicates a demand and request from the audience, and the absence of staring look at the spectator, which indicates a presentation.

##### The staring look at the spectator

**Figure 1:**
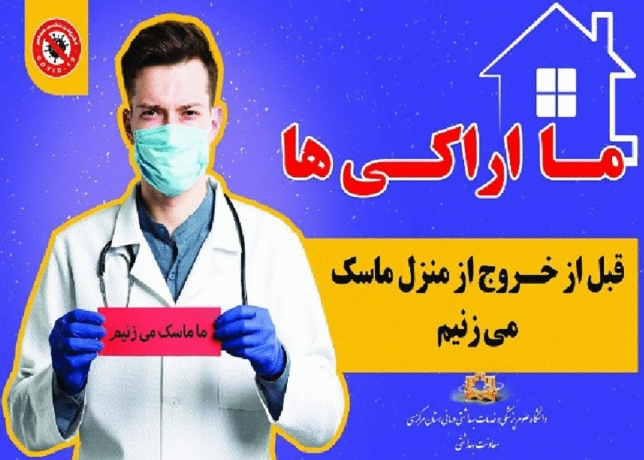
The staring look at the spectator.

In the Fig. 1, the content producer asks the audience to wear a mask before leaving the house. Therefore, to portray this concept and attract the audience, she/he has used the staring look at the spectator.

##### The absence of staring look at the spectator

**Figure 2:**
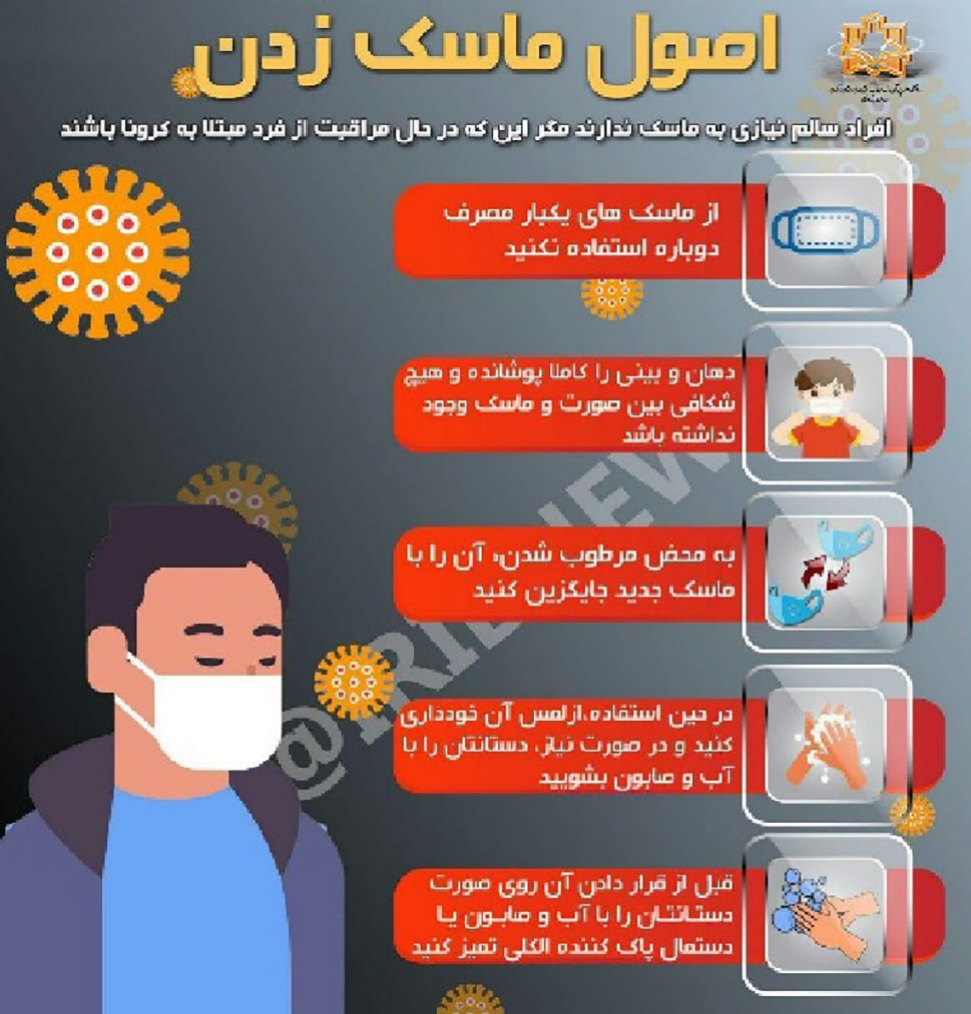
The absence of staring look at the spectator.

In the Fig. 2, the content producer intends to present and teach the correct method and principles of masking to the audience. Therefore, in order to better present the content to the audience, the method of the absence of staring look at the spectator is used so that the audience is attracted to the content presented.

#### Types of shot in pictures

The composition of the image and the position of the subject in the range of the frame seen by the camera is called the shot^54^. Therefore, the shot includes how the subject is placed in the image frame, the size of the subject that covers the surface of the image, and the angle that the camera has to the subject^54^. The types of shot are: close-up, medium shot, long shot^54^.

##### Close-up

**Figure 3:**
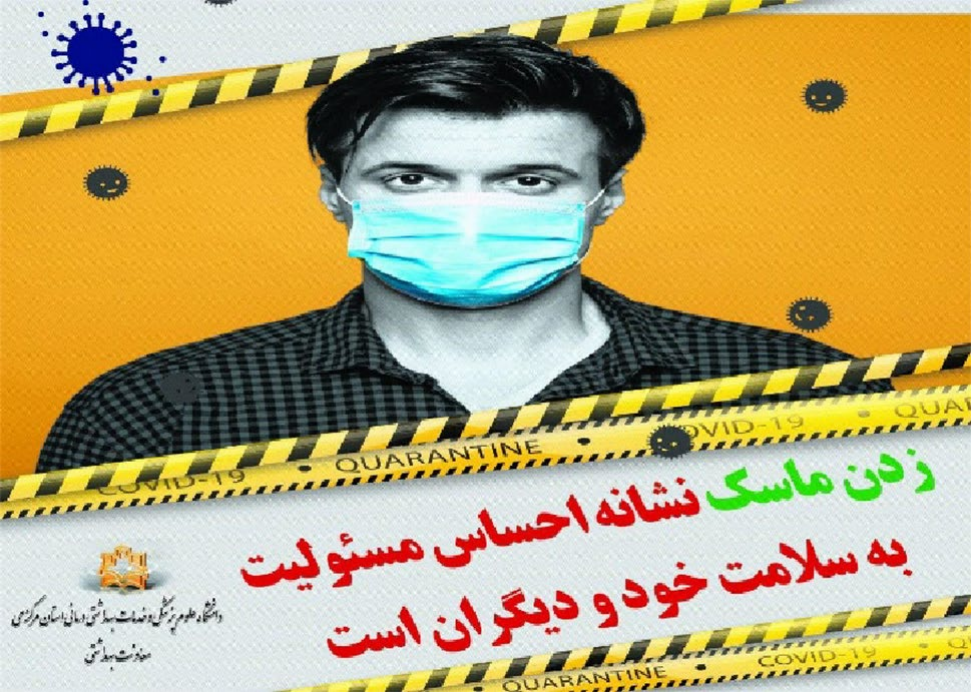
Close-up.

A close-up shows the details of the subject and accentuates the emotions^54^. In other words, close-up is used to portray concepts that involve personal and intimate relationships^54^. In the image below, a close-up of the subject, which represents personal and intimate relationships, along with a Dutch angle shot, which represents isolation, portrays the concept of “presentation” to the audience. In the Fig. 3, the inconsistency of the shot and camera angle with the desired concept causes the audience to fail to attract.

##### Medium shot

**Figure 4:**
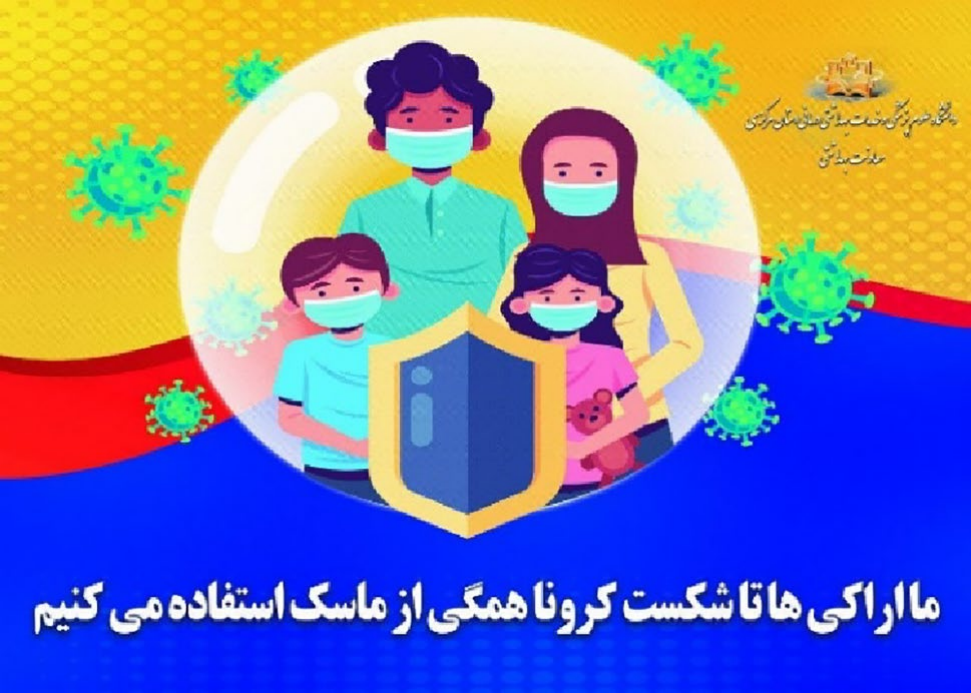
Medium shot.

In the medium shot, in addition to emphasizing the main subject, the surrounding environment of the subject is also depicted, which shows the creation of social relationships^54^. In the Fig. 4, the family, which is a social institution, is depicted in a frame and in a medium shot without showing the surrounding environment of the subject. Considering that the concept of the text indicates the creation of intimate relationships between the subject and the audience, presenting the desired concept in close-up will attract the audience better.

##### Long shot

**Figure 5:**
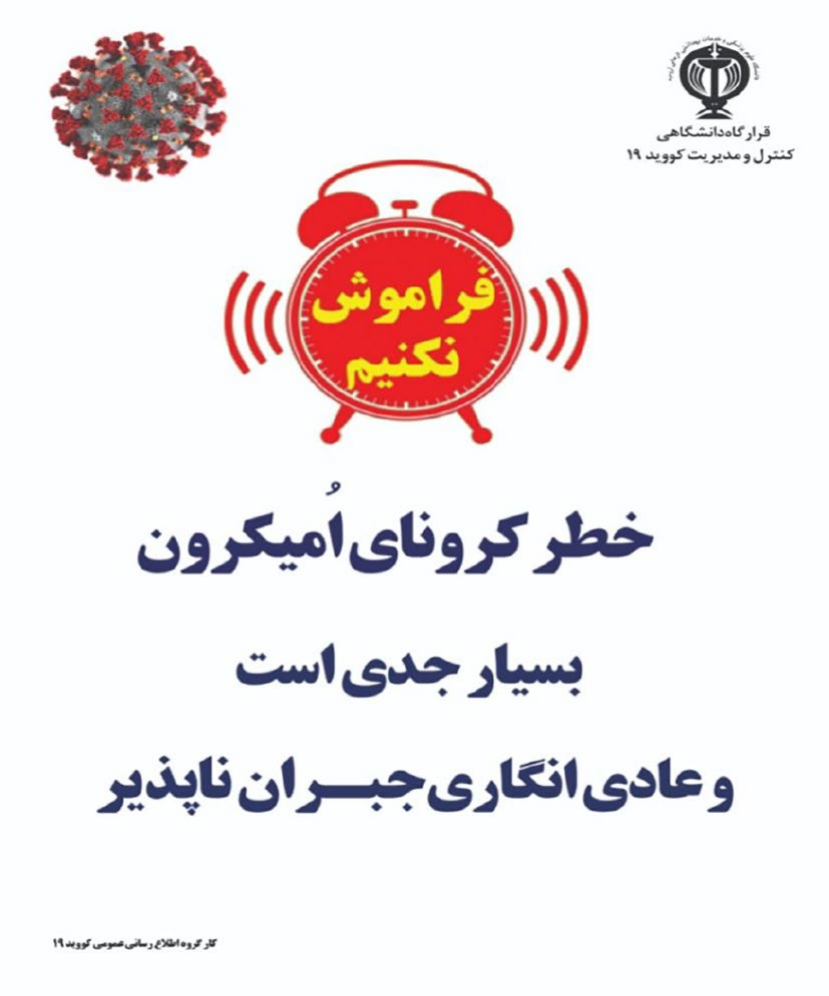
Long shot.

In the long shot, the subject is displayed from a distance and the content producer emphasizes the position and location^54^. Also, in the distant view, non-personal relationship is depicted^54^. In the Fig. 5, the desired subject, which is the image of the Coronavirus disease, is depicted from a long shot. Also, the desired subject is placed on the left side and on the top of the billboard. In terms of information value, the elements on the left are pre-existing and known elements, and the top of the billboard is the ideal information location^55,56^. Considering that the phrase in the image is related to the new strain of Coronavirus disease, i.e. Omicron, which is not a pre-existing and known element, therefore placing the subject in this position and in the long shot reduces the importance of the subject for the audience. As a result, it causes not attracting the audience and not encouraging them to follow health solutions to prevent the spread of the virus.

#### Types of shot angles in images

The shot angle is a way to describe the subject. The position of the camera angle relative to the ground creates different shots. Diversity in the angle of view creates different concepts that play an essential role in presenting the material to the reader. In other words, with the help of the camera angle, the importance and special status of the event and effective communication with the audience occurs^54^. In general, shots angles are divided into several main categories, which are: head-on shot, Dutch angle shot, high-angle shot, level shot, down shot^54^.

##### Head-on shot

**Figure 6:**
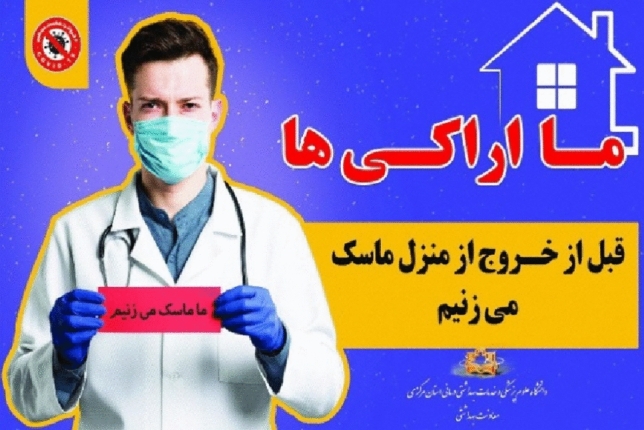
Head-on shot.

The head-on shot means that the audience is standing face to face with the subject^54^. In this case, the camera is placed directly in front of the subject’s eyes, and since this angle of view is usually used in human communication, it will induce a sense of intimacy and closeness between the audience and the subject. In fact, this state means equality and lack of feeling of superiority between the subject and the audience. In the Fig. 6, the head-on shot is used to express the concept of “we” and being together.

##### Dutch angle shot

**Figure 7:**
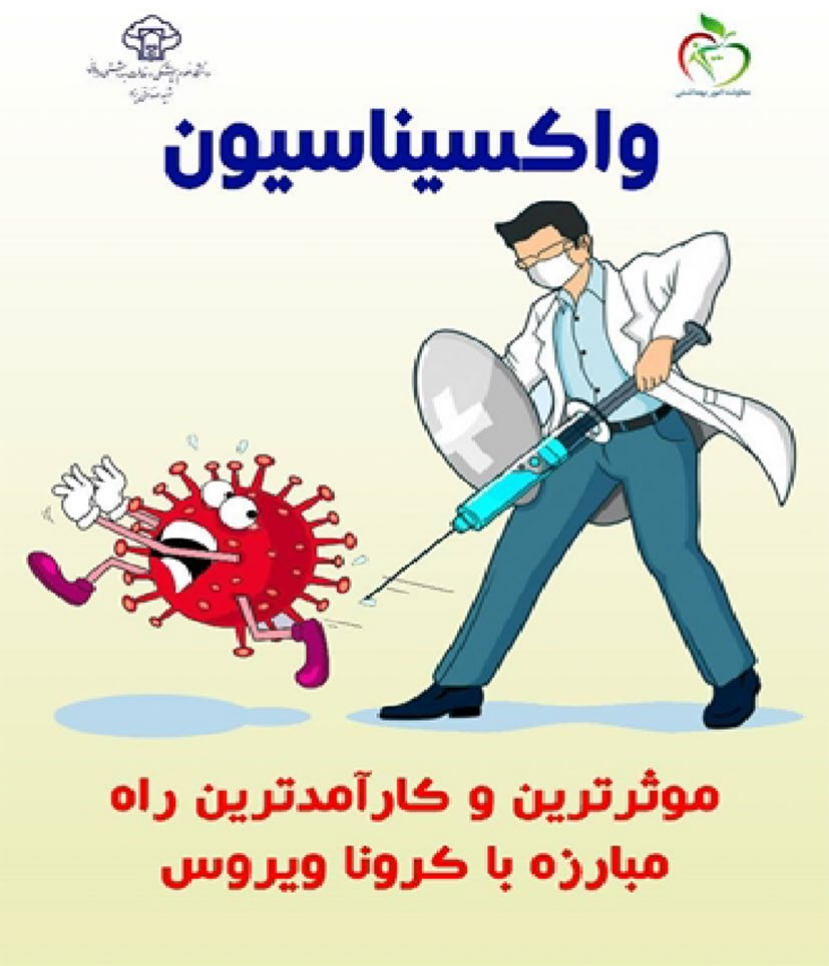
Dutch angle shot.

Dutch angle shot means looking at the subject crookedly^54^. This type of camera angle is often used to present concepts such as separation, war, imbalance, inner turmoil, hallucinations, explosions, discordant and disturbing events, worry, etc. to the audience^54^. In the Fig. 7, the concepts of combating and escaping the Coronavirus disease using a vaccine are depicted from Dutch angle shot.

##### High-angle shot

**Figure 8:**
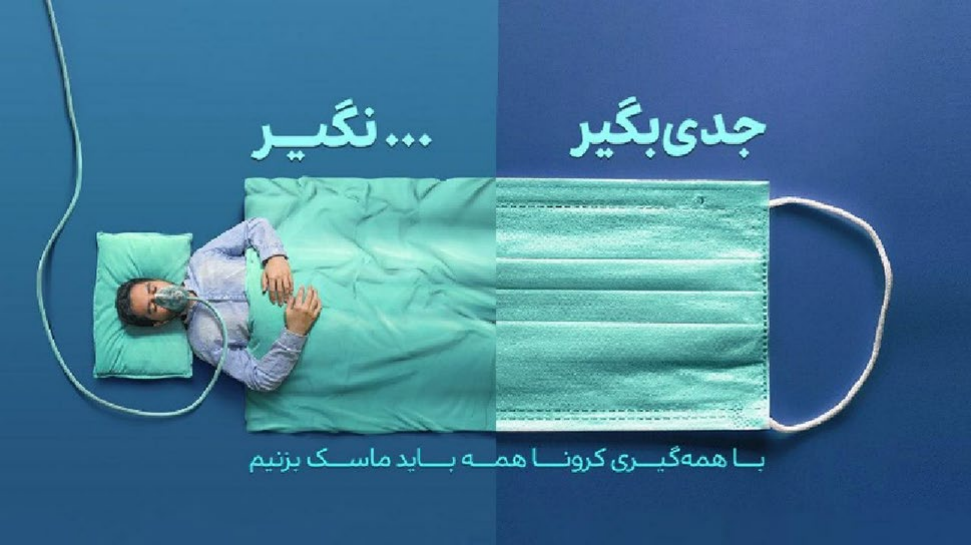
High-angle shot.

High-angle shot means looking from top to bottom. In this angle, the camera is pointing down, where the object or character is^54^. The high-angle shot has different degrees. In the Fig. 8, the camera angle is above the character’s head and depicts the subject vertically and 90°. In this type of camera angle, usually most of the shots and angles are made from the point of view of the character and the same size as humans, which sometimes gives a feeling of limitation to the audience, and for this reason, this type of camera angle represents power to the audience. In the Fig. 8, the content producer powerfully limits the audience to take the situation seriously and wear a mask and stay healthy, as opposed to not taking the situation seriously and not wearing a mask and illness, which ultimately creates unfavorable conditions for the individual.

##### Level shot

**Figure 9:**
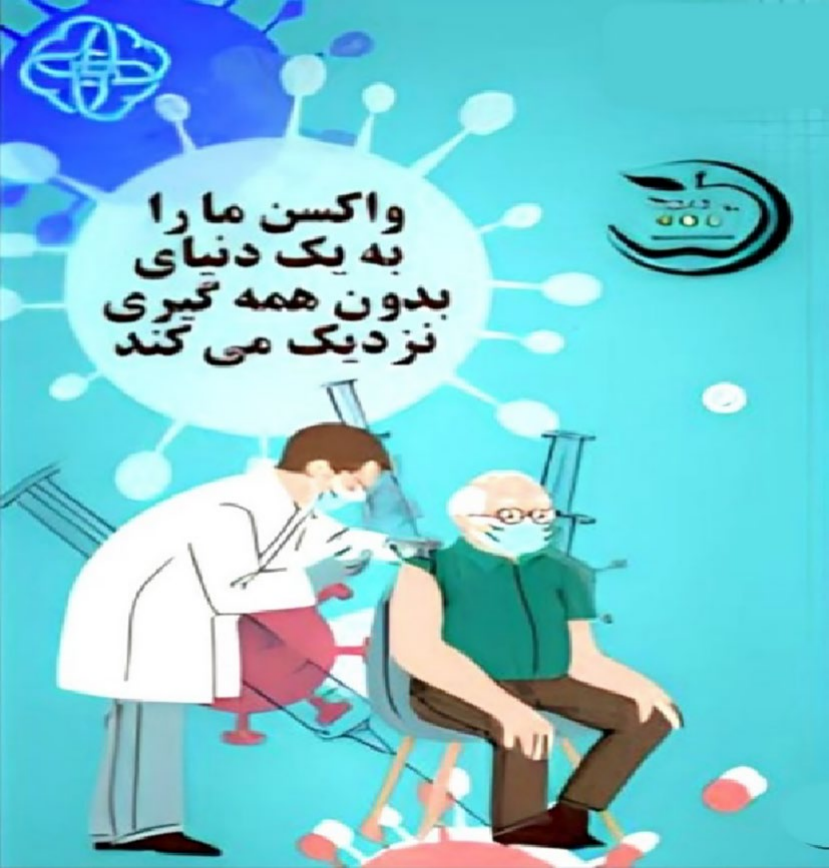
Level shot.

One of the most common camera angles and heights to show the content creator’s point of view is the level shot. In the level shot, the person or object represented is in its natural angle. Using the level shot is to express equality and remove the barrier between the audience and the produced content ^54^. The Fig. 9 shows the level shot. In the Fig. 9, the camera angle is the state where the height of the camera from the ground is equal to the height of the waist or the middle part of the subject’s body. This type of camera angle is used when one subject is sitting and the other is standing, and it is used to increase the tension or show the power of the subject.

##### Down shot

**Figure 10:**
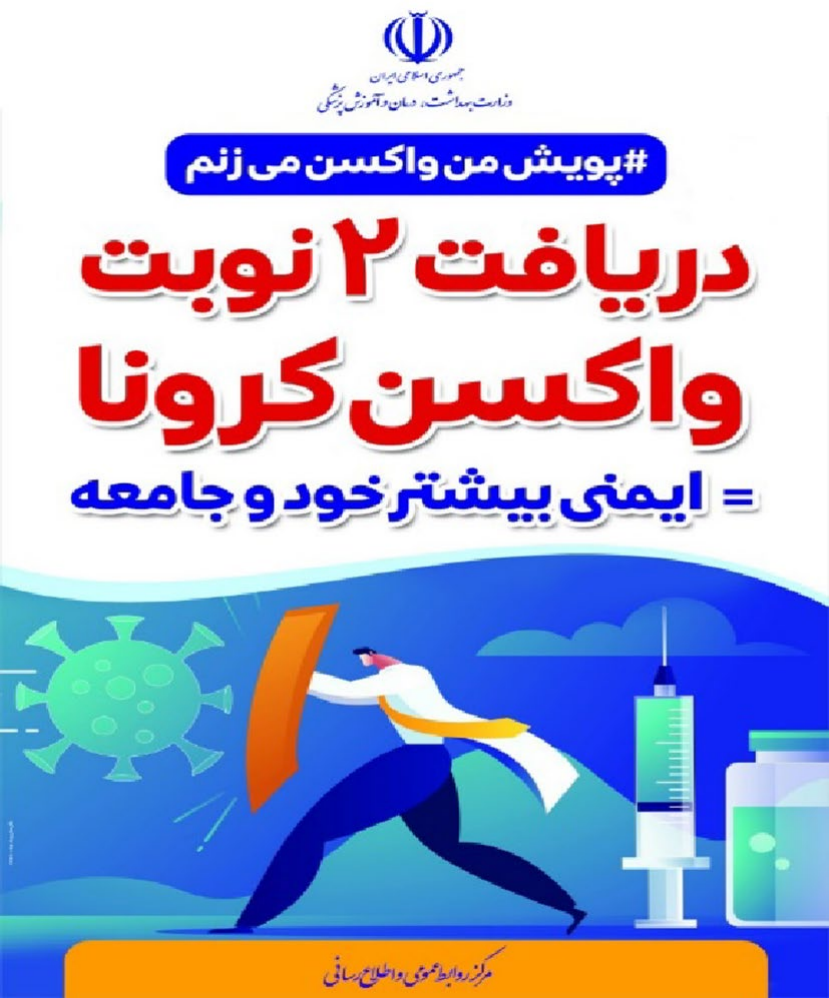
Down shot.

Down shot means looking from bottom to top. When the subject is seen from a down shot in the frame, so that the camera is positioned below the subject’s eye line, it indicates a dramatic space that generally emphasizes the dynamic force between the characters^54^. Like the scene of a hand-to-hand fight between two warriors^54^. Among the capabilities of the down shot is to show the strength and superiority of a character and also create a sense of participation for the represented person^54^. In the Fig. 10, the combat between the two characters of Coronavirus disease and the vaccine is depicted. In this combat, the vaccine is more powerful and it is the armor that protects people in the combating the Coronavirus disease and makes the virus escape.

### Statistical analysis

The current research aims to discover and identify the best visual factors presented in billboards and infographics related to Covid-19 in order to have a greater impact on the audience and encourage people to perform preventive behaviors and general vaccination. In this regard, exploratory analysis according to the purpose was carried out based on the valid approach of qualitative research and using the analysis of expert researchers. Next, the main question of the research is whether the expert-oriented approach about the best visual items is really aligned with the public opinion about the most attractive and effective visual items. Therefore, in order to investigate public opinion, quantitative method and descriptive statistics have been used so that the results of this research can be used in the real context of people’s lives and in accordance with the culture of the target society. In this section, people’s attitude towards pictures and their content is investigated using a survey that was distributed an online questionnaire, so that based on the highest frequency, the pattern of medical advertisements can be discovered and compiled. This study was conducted with the participation of 350 Iranian adults. The average age of the participants was41.94%. 199 (56.86%) of the participants were female and 151 (43.14%) were male. 224 (64%) were married, 95 people (27.14%) were single, 12 people (3.43%) were divorced and 19 people (5.43%) were widows. 48 people (13.71%) have free jobs, 59 people (16.86%) have government jobs, 15 people (4.29%) workers, 117 people (33.43%) were students, 24 people (6.86%) are housewives, 54 people (15.43%) are retired, 14 people (4%) were unemployed and 19 people (5.42%) had other jobs. Also, the number of 12 people (3.43%) low education, 40 people (11.43%) diploma, 114 people (32.57%) were bachelor’s degree, 83 people (23.71%) were master’s degree, 63 people (18%) were PhD and 38 people (10.86%) were postdoctoral.

As Table 3 shows, the analyzed data includes 36 images containing advertising texts, of which 17 (47.22%) images have a medium shot (creating a social relationship), 15 (41.67%) images have a dutch angle shot (separation), 11 (30.56%) images with a level shot (equality), 9 (25%) images with a head-on shot (inclusion), 8 (22.22%) images containing the absence of staring look at the spectator (presentation), the staring look at the spectator (demand) and close-up (intimate/individual relationship) both 7 cases with an average of 19.44%, 6 (16.67%) images with a down shot (creating a sense of participation for the represented person), 3 (8.33%) images with a long shot (creating a non-individual relationship) and 1 (5.56%) image have a high-angle shot (presenting power to the spectator).

**Table 3:** The frequency of Kress and Van Leeuwen model components in research data.

Elements in the image (conceptual representation)	Frequency	Percentage (%)
The staring look at the spectator (demand)	7 (of 36)	19.44
The absence of staring look at the spectator (presentation)	8 (of 36)	22.22
Close-up (intimate/individual relationship)	7 (of 36)	19.44
Medium shot (creating a social relationship)	17 (of 36)	47.22
Long shot (creating a non-individual relationship)	3 (of 36)	8.33
Head-on shot (inclusion)	9 (of 36)	25
Dutch angle shot (separation)	15 (of 36)	41.67
High-angle shot (presenting power to the spectator)	1 (of 36)	5.56
Level shot (equality)	11 (of 36)	30.56
Down shot (creating a sense of participation for the represented person)	6 (of 36)	16.67

Table 4 contains survey information on people’s interest in research data (billboards and infographics). The total number of people participating in this research is 350 people, of which 342 (97.71%) of the participants chose staring look at the spectator (demand). After that, in order from the highest to the lowest number, it is as follows: 337 (96.29%) people, head-on shot (inclusion); 315 (90%) people, down shot (creating a sense of participation for the represented person); 311 (88.86%) people, close-up (intimate/individual relationship); 291 (83.14%) people, level shot (equality); 259 (74%) people, high-angle shot (presenting power to the spectator); 165 (47.15%) people, the absence of staring look at the spectator (presentation); 143 (40.86%) people chose the dutch angle shot (separation); 94 (26.86%) people, medium shot (creating a social relationship) and 54 (15.43%) people chose the long shot (creating a non-individual relationship).

**Table 4:** The frequency of people’s attitude towards pictures and their content.

Elements in the image (conceptual representation)	Frequency	Percentage (%)
The staring look at the spectator (Demand)	342 (of 350)	97.71
The absence of staring look at the spectator (Presentation)	165 (of 350)	47.15
Close-up (intimate/individual relationship)	311 (of 350)	88.86
Medium shot (creating a social relationship)	94 (of 350)	26.86
Long shot (creating a non-individual relationship)	54 (of 350)	15.43
Head-on shot (inclusion)	337 (of 350)	96.29
Dutch angle shot (separation)	143 (of 350)	40.86
High-angle shot (presenting power to the spectator)	259 (of 350)	74
Level shot (equality)	291 (of 350)	83.14
Down shot (creating a sense of participation for the represented person)	315 (of 350)	90

## Discussion

The purpose of this research was to analyze the pictures of infographics and advertising billboards related to promoting preventive behaviors and taking measures to vaccinate the corona pandemic in the general adult population of Iran. The findings show that 47.22% of the advertisements of preventive behaviors and vaccination against the Coronavirus disease in Iran are presented in the medium shot and have the component of “creating a social relationship” in the theme. Meanwhile, the results of the survey show that more than half of the participants in this research (73.14%) believe that the medium shot (creating a social relationship) had no effect on attracting them to preventive measures. This lack of coordination between people’s attitude and the component presented in the images will have a bad effect on people’s behavior. Also, the results show that the images that contain the staring look at the spectator and have a demand component in the theme have attracted 97.71% of the people and have been able to establish a relationship with the audience visually. Meanwhile, 19.44% of the images presented the concept of “demand”, which is a small amount. Images with a head-on shot presenting the concept of “inclusion” attracted 96.29% of the audience and only 25% of billboards and infographics are designed with this type of camera angle, which is a very low average for content production considering the positive effect of this component. Images presented with a down shot and expressing the concept of participation have attracted 90% of people’s opinions, while only 16.67% of images with this type of camera angle have been produced and published. The images designed with a high-angle shot and presenting the concept of power, have allocated only 5.56% of all images, which is a very small percentage considering that this type of camera angle has attracted the opinion of 74% of people. Also, this result shows that applying power and looking from top to bottom is very important in presenting concepts related to preventive behaviors, and in addition to presenting concepts such as equality, intimacy, participation, etc., influence of power is necessary for the implementation of health regulations. 19.44% of the pictures have a close-up view and it attracted the opinion of 88.86% of people. This type of images, in a symbolic system, show subjects that are “a part of us” and create a “sense of participation” in people. On the other hand, 8.33% of the images have a long shot and 84.57% of people were not interested in such billboards and infographics. This result shows that presenting the concept of “impersonal relationship” did not have an effect on attracting people to perform preventive behaviors. Images with a dutch angle shot, which has the concept of separation in the theme, only attracted the opinion of 40.86% of the audience. This shows that the separation component does not have a great effect on motivating people to engage in preventive behaviors. The images that are presented with the same level shot and in the concept of equality, have attracted 83.14% of the audience, while only 30.56% of the images were produced and designed with this type of camera angle. Images containing the absence of staring look at the spectator, which has the concept of “presentation” in the theme, also attracted 47.15% of people. This shows that the concept of “presentation” for preventive behaviors during the Coronavirus disease epidemic has been useful, but it has not been able to motivate many audiences towards the implementation of the provided health strategies and guidelines. Therefore, such concepts can be designed in a combined manner with other components. For example, the design of billboards and infographics containing the absence of staring look at the spectator along with close-up that create a sense of individual intimacy in people, can be useful in advertisements related to providing preventive solutions during the epidemic.

Thus, the results of this research show that teaching health tips and communicating effectively in the field of health, especially during an epidemic like Corona, should be in line with the way of thinking and culture of ordinary people to transfer messages better, faster and more effectively. These results, in addition to being in accordance with the theory and methodological approach of this research^51^, it is also in line with previous researches^14–16,41–43^. What is important in this context is to identify these effective patterns in order to use them as best as possible. Also, the results show that advertising in the field of preventive measures in order to encourage people to carry out the desired activities in the field of medical sciences requires special rules that determine the culture of people and the main foundation of their attitude and thinking. These results are also in line with the researches of Katermina and Lipiridi^39^ and Cliff and Anna^36^. Attracting public trust by using knowledge-based visual signs by responsible institutions in the field of public health in acute conditions such as Covid-19 acts as a cognitive shortcut that increases people’s understanding of an unknown and uncontrollable risk. As a result, these images effectively attract the attention of ordinary people and non-experts to perform preventive behaviors in epidemic control and facilitate the communication between people and scientists in the field of health. These results are in line with Li & Molder’s research^14^. Therefore, the production of knowledge-based advertising infographics and billboards based on scientific principles in the field of coronavirus prevention are very effective in raising the awareness of people in the community to deal with the epidemic and help to understand the messages conveyed through visual communication strategies. These results are in line with the researches of Li & Molder’s^14^, Shemies^15^, Taif^42^ and Sombilon^16^. Therefore, it can be concluded that in visual communication, visual components, like linguistic components, play a significant role in creating and maintaining desired ideologies, and this role can serve to create, perpetuate and legitimize concepts. The results also show that in visual communication, visual components, like linguistic components, play a significant role in creating and maintaining desired ideologies, and this role can serve to create, perpetuate and legitimize concepts. Therefore, the presence of misleading visual cues can lead to miscommunication with long-term negative impact. These results are in line with Lund^34^ and Bonvillain^35^ and Li & Molder’s^14^.

**Figure 11:**
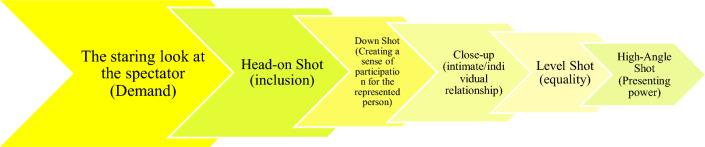
The model presented to provide health regulations and preventive measures during the Coronavirus disease epidemic.

Therefore, based on the results, the Fig. 11 model can be presented to provide health regulations and preventive measures during the Coronavirus disease epidemic in order to receive useful feedback from people in the society.

## Conclusion

With the increasing predominance of visual culture in contemporary times, it is extremely important to pay attention to the methods of analyzing images and visual texts. The purpose of this research is to investigate and analyze the images of advertising infographics and billboards related to the promotion of preventive behaviors and vaccination of the Coronavirus disease pandemic, as well as to investigate the opinion of the general adult population of Iran regarding the components in the images. In general, based on the results of this research, it can be concluded that visual factors are very effective in attracting the audience in order to persuade them to implement health messages. Therefore, when teaching health and preventive principles, the correct and effective use of visual factors for maximum effectiveness is important. So that if an effective communication pattern is used and is in line with the way of thinking of the audience based on scientific principles, the main message will be transmitted faster, easier and more effectively. This issue becomes more apparent, especially during the outbreak of an epidemic (such as during the Covid-19 epidemic) when universal and public preventive measures gain special value and importance. As the results of the present study also showed its importance in this regard. What is important is that there have been epidemics throughout human history, and the coronavirus will not be the first or the last. Therefore, conducting scientific research during the outbreak of this virus can be an opportunity to solve the challenges in this field. In general, as the results of the present study also showed, billboards and infographics are one of the most important advertising tools in the field of health due to their availability to all members of society. In this regard, scientific principles based on research can be used to increase attractiveness, the speed of message transmission and increase the number of audiences. As the present study also aimed to achieve the same principles. Among the other achievements of the results of this research is the presentation of a final model of multimedia content tailored to Iranian culture with maximum effectiveness on the general population of Iran. Also, considering that the target population in Iran is all strata of the society; therefore, the results of the current research, in addition to promoting preventive behaviors and vaccination of the Coronavirus disease pandemic, are used as a more general model to promote the improvement of the health culture of the society. Thus, the findings and results of this research can help all public health officials and medical education centers to manage and control the crisis in a fully professional and planned manner in case of possible similar crises and communicate better with people about public health.

## Limitations and future research

Using Cress and Van Leeuwen discourse analysis model, this research has provided a new and systematic framework for the qualitative evaluation of the pictures of billboards and infographics in the field of prevention of the coronavirus epidemic in Iran. Considering that this research is one of the first researches that tried to provide a practical model in the field of disease prevention advertisements, therefore, it is in the early stages and more investigations should be done based on the components presented in this field so that a global model in this field can be achieved based on it. According to the surveys, Iran is facing a lack of research related to advertising in the field of medical sciences. From this point of view, it is necessary to carry out research in the field of medical science advertising based on the interpretation of the macro structures of Iranian society and to discover the appropriate model to present such issues. Also, languages have principles and parameters. The principles exist in all languages and are universal, but the parameters are the linguistic differences that have caused a particular language to emerge and differ from one language to another. Languages reflect the culture of societies. Therefore, it is suggested that research in the field of disease prevention advertisements be conducted on the languages and cultures of different societies so that a comprehensive and complete model in this field can be obtained from the results of these studies.

## Data Availability

All data analyzed during this study are included in this published article. A number of billboards and infographics had been installed in the city, which were photographed using a camera. A number of images have also been received from reliable sites whose links are mentioned below. https://omana.mui.ac.ir/fa/node/941. https://jajarm.nkums.ac.ir/Content/76761/%D8%AC%D8%AF%DB%8C-%D8%A8%DA%AF%DB%8C%D8%B1. https://farsi.khamenei.ir/photo-album?id=46122. https://www.irefco.ir/%D9%BE%D9%88%D9%8A%D8%B4_%D9%85%D9%84%D9%8A_%D9%85%D9%86_%D9%85%D8%A7%D8%B3%D9%83_%D9%85%D9%8A%D8%B2%D9%86%D9%85/. https://www.ifsm.ir/photo/51166/%D9%85%D8%A7%D8%B3%DA%A9-%D9%86%D8%AC%D8%A7%D8%AA-%D8%A8%D8%B2%D9%86%DB%8C%D8%AF. https://arakmu.ac.ir/vch/fa/page/6463/%D9%BE%D9%88%DB%8C%D8%B4-%D8%B2%D8%AF%D9%86-%D9%85%D8%A7%D8%B3%DA%A9. https://rasekhoon.net/photogallery/show/1514662/%D9%85%D8%A7%D8%B3%DA%A9-%D9%86%D8%AC%D8%A7%D8%AA. https://www.khabaronline.ir/photo/1504611/%D8%A8%DA%A9%D8%B4-%D8%A8%D8%A7%D9%84%D8%A7-%D8%A7%D9%88%D9%86-%D9%85%D8%A7%D8%B3%DA%A9-%D9%84%D8%A7%D9%85%D8%B5%D8%A8-%D8%B1%D9%88. https://www.tasnimnews.com/fa/media/1400/04/30/2541887/%DA%A9%D8%A7%D8%B1%DB%8C%DA%A9%D8%A7%D8%AA%D9%88%D8%B1-%D8%AA%D8%B9%D8%B7%DB%8C%D9%84%DB%8C-%D9%BE%D8%A7%DB%8C%D8%AA%D8%AE%D8%AA-%D8%A8%D9%84%D8%A7%DB%8C-%D8%AC%D8%A7%D9%86-%D8%B4%D9%87%D8%B1%D9%87%D8%A7%DB%8C-%D8%B4%D9%85%D8%A7%D9%84%DB%8C-%DA%A9%D8%B4%D9%88%D8%B1-%D8%AA%D8%B1%D8%AF%D8%AF-%D8%B1%D9%88%D8%A7%D9%86-%DA%A9%D8%B1%D9%88%D9%86%D8%A7-%D8%A8%DB%8C%D9%86-%D8%AA%D9%87%D8%B1%D8%A7%D9%86-%D8%B4%D9%85%D8%A7%D9%84. https://www.mehrnews.com/news/5342795/%D9%88%D8%A7%DA%A9%D8%B3%D9%86-%D8%A8%D8%B2%D9%86%DB%8C%D8%AF-%D8%AA%D8%A7-%D8%B2%D9%86%D8%AF%D9%87-%D8%A8%D9%85%D8%A7%D9%86%DB%8C%D8%AF. https://www.yjc.ir/fa/news/7916065/%D8%B7%D8%B1%D8%AD-%D8%A8%D8%B1%D8%A7%DB%8C-%D9%87%D9%85-%D8%A8%D9%85%D8%A7%D9%86%DB%8C%D9%85%D8%B7%D8%B1%D8%AD%DB%8C-%D8%A8%D8%B1%D8%A7%DB%8C-%D8%AA%D8%B4%D9%88%DB%8C%D9%82-%D8%B4%D9%87%D8%B1%D9%88%D9%86%D8%AF%D8%A7%D9%86-%D8%A8%D9%87-%D9%88%D8%A7%DA%A9%D8%B3%DB%8C%D9%86%D8%A7%D8%B3%DB%8C%D9%88%D9%86-%D8%B9%D9%84%DB%8C%D9%87-%DA%A9%D8%B1%D9%88%D9%86%D8%A7. https://www.isna.ir/news/1400071107535/%D8%A8%D8%A7-%D9%88%D8%A7%DA%A9%D8%B3%DB%8C%D9%86%D8%A7%D8%B3%DB%8C%D9%88%D9%86-%DA%A9%D8%B1%D9%88%D9%86%D8%A7-%D8%A8%D8%B1%D8%A7%DB%8C-%D9%87%D9%85-%D8%A8%D9%85%D8%A7%D9%86%DB%8C%D9%85. https://www.ifsm.ir/photo/52897/%D9%85%D9%88%D8%AB%D8%B1-%D8%AA%D8%B1%DB%8C%D9%86-%D9%88-%DA%A9%D8%A7%D8%B1%D8%A2%D9%85%D8%AF%D8%AA%D8%B1%DB%8C%D9%86-%D8%B1%D8%A7%D9%87-%D9%85%D9%82%D8%A7%D8%A8%D9%84%D9%87-%D8%A8%D8%A7-%DA%A9%D8%B1%D9%88%D9%86%D8%A7-%D9%88%DB%8C%D8%B1%D9%88%D8%B3. https://www.alef.ir/news/4000523025.html?show=text. https://arakmu.ac.ir/farahandh/fa/news/28100/%D9%88%D8%A7%DA%A9%D8%B3%DB%8C%D9%86%D8%A7%D8%B3%DB%8C%D9%88%D9%86-%D9%87%D9%85%DA%AF%D8%A7%D9%86%DB%8C-%D9%85%D9%88%D8%AB%D8%B1%D8%AA%D8%B1%DB%8C%D9%86-%D8%B1%D9%88%D8%B4-%D8%AF%D8%B1-%D8%AA%DA%A9%D9%85%DB%8C%D9%84-%D9%85%D8%A8%D8%A7%D8%B1%D8%B2%D9%87-%D8%A8%D8%A7-%DA%A9%D9%88%D9%88%DB%8C%D8%AF%DB%B1%DB%B9. https://health.bpums.ac.ir/Fa/DynPages-8977.htm.

## Abbreviation

COVID-19: Coronavirus disease
